# Transcriptomic Analysis of Phylloclade in *Ruscus aculeatus* Is Consistent with Unifacial Morphology

**DOI:** 10.3390/plants15081168

**Published:** 2026-04-10

**Authors:** Edward M. Golenberg, Aleksandar Popadić, Weilong Hao

**Affiliations:** Department of Biological Sciences, Wayne State University, Detroit, MI 48202, USA; apopadic@wayne.edu

**Keywords:** adaxial, abaxial, development, planar development, laminar growth, transcriptome, differential gene expression, phylloclades, monocots, *Ruscus aculeatus*

## Abstract

The development of planar structures such as wings or leaves is a common feature among organisms and serves as a mechanism to increase surface to volume ratios. We wished to explore whether the recurrent and independent development of similar adaptive planar morphologies is the result of an activation of common genetic modules or toolkits. To test this, we focused on the developmental gene networks that are proposed to define leaf polarity in eudicots in phylloclades, leaf-like organs derived from branch primordia, in the monocot Ruscus aculeatus. Since branch primordia normally have a radial shape, this approach allowed us to examine the genetic changes required for the transformation from a round to a planar (flat) form. In our transcriptome analysis of phylloclade and stem tissue, we detected 76,085 annotated ORFs of which 87.2% were identified as complete out of 2026 BUSCO groups. Expression patterns clearly identify differentiation between phylloclade and stem tissues consistent with an enhanced photosynthetic function in the phylloclades. However, except for the *AS1*/*AS2* and possibly *STM* module, we see little evidence that canonical leaf adaxial and abaxial modules are activated in the sampled phylloclades compared with the stems. Our results show that the unifacial nature of phylloclades is consistent with the observed lack of strong adaxial/abaxial molecular signatures. We propose that in R. aculeatus and plants with similar unifacial laminar leaves, adaxial/abaxial molecular identity may not be required for planar growth, and that lateral expansion of organ primordia and acropetal and intercalary cell division may be sufficient to generate planar versus radial organ shapes.

## 1. Introduction

The independent evolution of functionally similar morphological or physiological structures and traits that mediate an organism’s response to environmental stress is known as convergent evolution. For example, in plants, independent origins of water-retaining tissues in leaves, stems and roots are common adaptations for growth in arid environments. Similarly, similar modifications of leaves or stems into hardened, sharp structures have evolved as an anti-herbivory strategy. Convergent evolution has traditionally been viewed as a hallmark of adaptive natural selection. Yet two related questions about the mechanisms of convergent evolution still arise: “Why is the adaptive landscape so restricted that similar fitness optima are reached?” and “How do distantly related species attain similar solutions?” In the case of embryophytes, it is thought that modern land plants are derived from a single lineage [1]. Thus, even with lineage-specific novel gene evolution, clades of extant organisms share a large portion of their genome, generally the more extensive the more recent the origin of the specific clade. As independent lineages evolve, the common genomic component, or “shared genetic toolkit” may be differentially used or inactivated. However, under similar selective pressures, reactivations or similar modalities of expression of these toolkits may result in similar phenotypes, fueling the “independent” evolution of convergent traits. As such, the study of convergent traits on a genomic and transcriptomic level can provide deeper insights into the existence and composition of such hypothetical “genetic toolkits” and into whether such traits are truly convergent or evolutionary reversals.

In the case of embryophytes, flat organs extending from the cylindrical stem and oriented roughly perpendicular to the sun have evolved several times independently [2]. The structures of these organs generally increase the area whereby sunlight and gases, particularly CO_2_, can be captured. The high surface to volume ratio allows for efficient distribution of the gases within the structure and release of heat, while decreasing the weight of the structure, allowing for greater extension and reduced torque. The most common such planar structures are megaphylls or leaves found in vascular plants, although other flatten structures have evolved from stems or branches such as cladodes and phylloclades.

**Figure 1:**
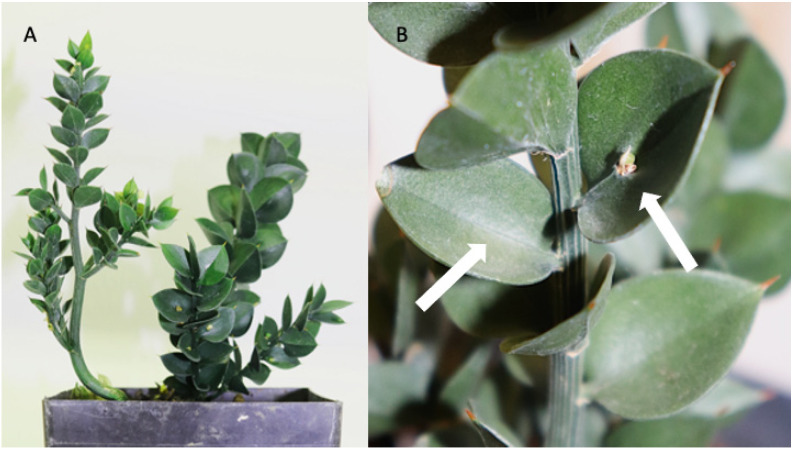
*Ruscus aculeatus* “Elizabeth Lawrence”. (**A**) View of whole plant. Multiple fertile phylloclades bearing flowers are visible. (**B**) Close up of phylloclades on a single stem. Arrows indicate sterile phylloclade (left) and fertile phylloclade (right). The fertile phylloclade bears a bract, and a flower situated above the midrib approximately a third of the length from the bottom of the phylloclade.

To explore whether fundamental genetic networks involved in the development of planar structure are co-opted to generate laminar structures from non-leaf origins, we focused on gene expression patterns in the phylloclades, flattened, determinate stems, found in *Ruscus aculeatus*. *R. aculeatus* is an evergreen, monocot, shrub with a Mediterranean and southwestern European distribution extending into southern England [3]. It is notable for its development of highly reduced, scalar leaves and spiny organs, referred to as phylloclades, cladophylls, or cladodes [3,4,5,6,7]. In this species, phylloclades serve as the primary photosynthetic organs with physiological adaptations for shade and drought tolerance [8]. These organs are either sterile or fertile, the latter of which bear a bract, flower, and ultimately a fruit on either the adaxial or abaxial surface (Figure 1) [4,7]. As the phylloclade is structurally and functionally analogous to a leaf, and as it is derived from an axial primordium that generally generates radially symmetric lateral shoots or branches, we wished to explore whether a leaf-like gene polarity toolkit is co-opted in this organ to convert the expected indeterminate branch into a determinate laminar appendage.

The composition of such leaf genetic toolkits has been dissected through the study of mutations in several transcription factor genes that resulted in needle-like leaves and cylindrical vascular steles [9,10,11,12] and subsequent studies of gene expression patterns and regulatory crosstalk. These studies led to the concept that adaxial (facing the stem or axis, dorsal)/abaxial (facing away from the stem, ventral) polarity is established by the expression domains of a defined suite of adaxial (e.g., *ASI/AS2, PHB, REV*) and abaxial (e.g., *KAN*, *ARF3/4*) genes [13,14]. Based on the observation that mutants that lacked abaxial or adaxial identity in leaves resulted in radially symmetrical structures, it is hypothesized that laminar development is a result of the juxtaposition of adaxial and abaxial gene expression leading to a planar outgrowth [12]. Due to their mutually antagonistic regulatory effects, adaxial and abaxial domains do not intersect. The hypothesis, then, is that the regions of contact at the leaf margin will result in novel gene regulatory networks that support a laminar (medial polar) growth rather than growth in a dorsal/ventral plane.

The objective of this study is to test whether the expression of adaxial and abaxial networks can explain the development of the phylloclades in *R. aculeatus*. Simply, does *R. aculeata* co-opt the adaxial and abaxial network to convert an indeterminate cylinder into a leaf-convergent, determinate flat organ? We analyzed transcriptomes from young phylloclade margin and center tissues and from internode stem tissue to identify canonical adaxial and abaxial genes and to compare their relative expression levels at one point in development time. We found that major classes of adaxial and abaxial identity genes are expressed in *R. aculeatus*. Contrary to expectations, expression patterns were lower or not detected in the phylloclades compared to expression in the stem internodes. These results, along with the morphology and development of the phylloclades, are consistent with morphological data suggesting that no strong adaxial/abaxial differentiation or bifacial identities persist in these organs and that, while planar, the organs are unifacial, having only a single, generally abaxial, identity. These results suggest that in *R. aculeatus* and plants with similarly unifacial laminar leaves, adjacent abaxial and adaxial domains may not be required for planar organ development, and that lateral expansion of organ primordia and acropetal and intercalary cell division may be sufficient to generate flattened versus radial organ shapes.

## 2. Results

### 2.1. Generation and Assembly of Ruscus aculeatus Transcriptome Data

To survey patterns of differential gene expression among phylloclade and stem tissues in *Ruscus aculeatus,* we generated de novo transcriptome data from each source tissue. The constructed reference assembly has 207,116 contigs with 169,563,644 bp in length. Its N50 is 1530 bp, and there are 76,085 annotated ORFs. A summary of sequence data statistics is listed in Table 1. BUSCO analysis (v6.0.0) performed on the transcriptome assembly sequences using the embryophyta_odb12 lineage dataset identified 87.2% complete, 7.9% fragmented, and 4.9% missing BUSCOs out of 2026 groups, indicating high coverage. Volcano plots showing log2 of expression ratios by phylloclade center and marginal tissue sources and phylloclade and stem tissues are shown in Supplemental Figure S1. There are 170 center-margin genes with an adjusted *p*-value (padj) < 0.05 for significantly different expression patterns (DEGs). Among the 170 center-margin DEGs, 60 DEGs with a robust expression level and fold change (baseMean > 50, log2FoldChange > 1) were used for further functional gene ontology (GO) analysis (details in Supplemental Table S1).

### 2.2. Distinct Gene Expression of DEGs in Center Phylloclade

**Figure 2:**
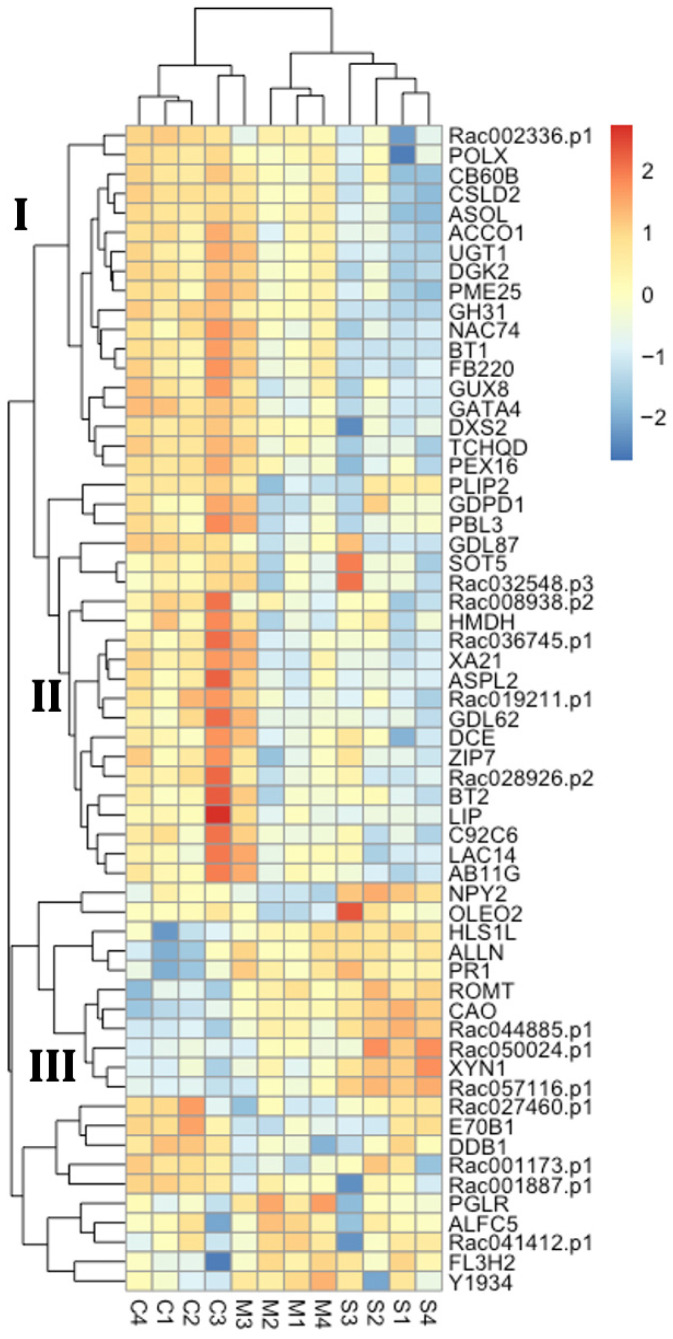
Clustering of gene expression in 60 center-margin DEGs that have a robust expression level and fold change between center and margin phylloclade tissues. Gene expression in phylloclade center, margin and stem tissues of these center-margin DEGs is presented. The rows represent identified gene paralogues in *Ruscus*. The columns represent the individual tissue samples; S1–4 are stem sample replicates, M1–4 are phylloclade margin sample replicates, C1–4 are phylloclade center replicates. Y axis hierarchical groupings reflect correlation of gene expression across tissues, while the X axis hierarchical groupings reflect correlation of sample expression patterns across genes. Genes are classified into three groups (I, II, III) based on their expression patterns. The continuous color scale in the heatmap represents relative expression values.

To understand the expression patterns of these 60 center-margin DEGs better, we present the clustering patterns of their expression in phylloclade center, phylloclade margin, along with stem tissues (Figure 2 and Figure S1a). The dendrogram at the top of the figure clusters sample transcriptomes by expression pattern affinities. The phylloclade center tissues are generally most distinct from the other tissue expressions, as expected given that the data are from center-margin DEGs. The single exception is the pairing of Center tissue sample C3 with Margin tissue sample M3, both of which were isolated from a single phylloclade. Along the side of the heat map, the dendrogram clusters genes by their expression patterns across samples. The clustering patterns display at least three consistent patterns. In clade I, 18 genes show higher expression in center phylloclade tissues and slightly lower expression in the phylloclade margins, but lower expression in stem tissues. Clades II (21 genes) and III (9 genes) indicate higher or lower expression levels in phylloclade center tissue compared to both phylloclade margin and stem tissues, respectively. Thus, phylloclade center tissues show different expression patterns compared to stem tissue patterns in 48 of the listed genes. In terms of this subset of genes, phylloclade margins could be distinguished from center tissue in 30 of these genes.

**Figure 3:**
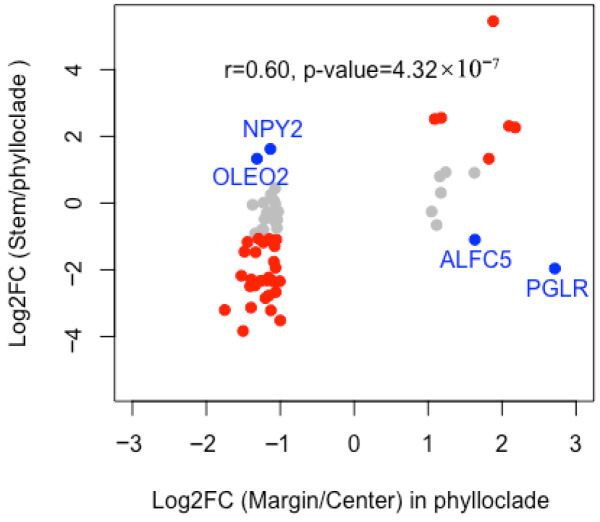
Comparison of the differential expression of the center-margin DEGs between the ratio of margin over center phylloclade versus the ratio of stem over phylloclade (i.e., combined margin and center phylloclade). Genes with correlated ratios (*n* = 34) are in red, genes with antagonistic ratios (*n* = 4) are in blue with gene names, and genes with fold change < 2 (|log2FC| < 1) (*n* = 22) are in gray. The correlation of all 60 ratios is r = 0.60 with a *p*-value = 4.32 × 10^−7^.

We sought to understand the source of the differential expression of center-margin DEGs, and conducted a correlation analysis of ratios of phylloclade margin to center and stem to total phylloclade expression patterns (Figure 3). There is a significant correlation (r = 0.60, *p*-value = 4.32 × 10^−7^) between margin-to-center expression ratios and stem-to-phylloclade expression ratios. Only four out of the 60 DEGs, *NPY1*, *OLEO2*, *AFLC5* and *PGLR*, show antagonistic ratios between margin-to-center expression and stem-to-phylloclade expression. Overall, center phylloclade is viewed to have the most distinct gene expression when compared against margin phylloclade and against stem.

### 2.3. Gene Ontology (GO) of the Center-Margin DEGs

**Figure 4:**
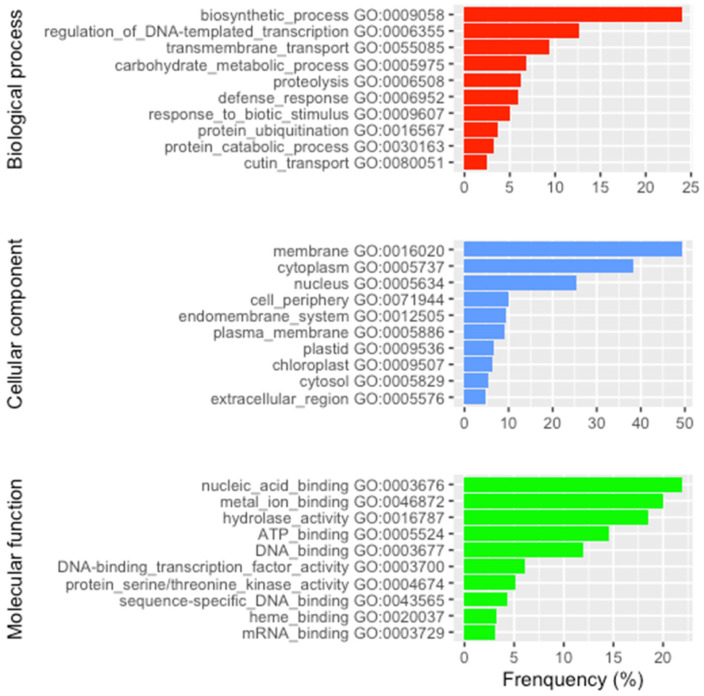
Distribution of the GO terms of the center-margin DEGs. Sample frequencies of the top 10 GO terms in each GO domain (biological process in red, cellular component in blue, and molecular function in green) are shown.

To explore whether the differences in expression pattern reflect a physiological differentiation between the stem and the phylloclade, we analyzed the GO terms that are significantly overrepresented in terms of their representative functionality. Under biological processes (Figure 4), the most common GO grouping is biosynthetic processes, followed by transcription regulation, membrane transport, and carbohydrate metabolism. Among the overrepresented cellular components category (Figure 4), plastid membrane, chloroplast, and membrane are among the top ten. Lastly, under Molecular Function (Figure 4), metal ion binding, ATP binding, heme binding, and hydrolase activity are among the most overrepresented categories. These groupings are consistent with a proposed enhanced photosynthetic role of the phylloclade compared to the stem.

### 2.4. Differential Expression Between Phylloclade and Stem and Variation Among Phylloclade Individuals

**Figure 5:**
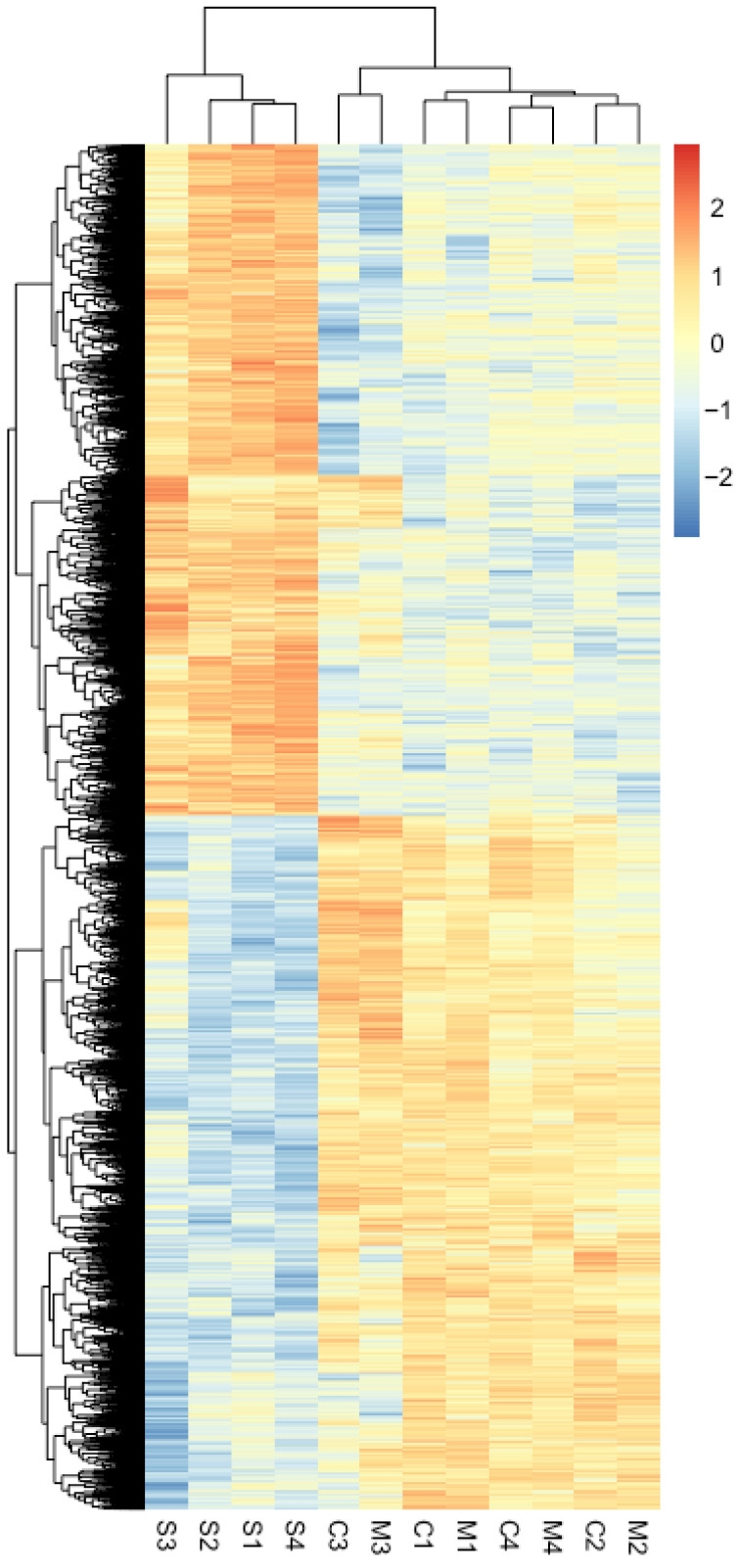
Clustering of 7238 genes that have differential expression between stem and phylloclade. The padj-value cutoff was required to be 0.01, given the large number of differentially expressed genes between stem and phylloclade. The rows represent identified gene paralogues in *Ruscus*. The columns represent the individual tissue samples; S1–4 are stem sample replicates, M1–4 are phylloclade margin sample replicates, C1–4 are phylloclade center replicates. Y axis hierarchical groupings reflect correlation of gene expression across tissues, while X axis hierarchical groupings reflect correlation of sample expression patterns across genes. The continuous color scale in the heatmap represents relative expression values.

Given the apparent functional distinction between the phylloclade and stem tissues, we analyzed genes showing differential expression between the stem and combined phylloclade tissues (Figure 5 and Figure S1b). The heat map shows a clear distinction between over-expressed and under-expressed stem transcripts in comparison with those of the phylloclade. All phylloclade tissues form a distinctly differentiated group from all stem tissues based on expression patterns. Yet, each phylloclade sample can be identified in comparison with the other. Such differentiation does not reflect random sampling variation among biological replicates but rather suggests that each organ is differentiated in terms of development, age, and/or physiological activity at the time of sampling.

### 2.5. Variation in Gene Presence/Absence and Paralog Copies in Developmental Axes Genes

**Figure 6:**
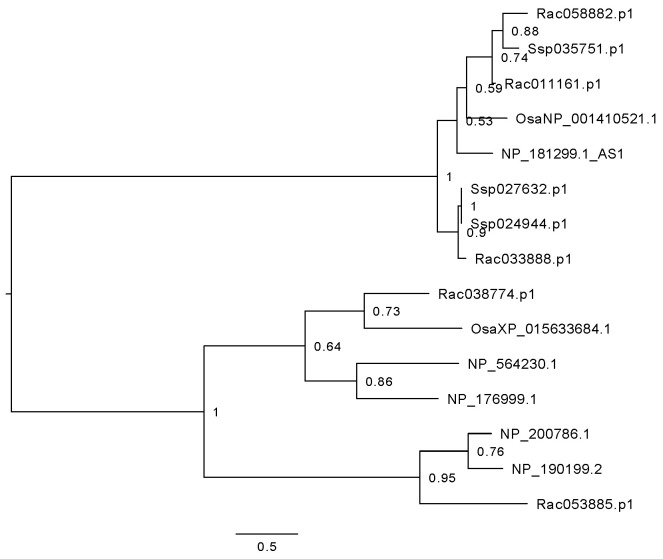
Phylogenetic tree of *AS1* gene families in *Arabidopsis thaliana* (gene name added after the accession number), *Oryza sativa* (Osa)*, Sansevieria subspica* (Ssp), and *R. aculeatus* (Rac). A three-letter species abbreviation is added at the beginning of each gene except for *Arabidopsis thaliana.* Bootstrap values are shown in percentages.

Gene networks in developmental axes have been recognized to play an important role in the development of eudicot leaves [13,15,16,17]. Based on the remarkably similar morphological and functional analogy between the phylloclade and true leaf, we hypothesized that an analogous developmental gene network would be invoked in the phylloclade as to that found in eudicot leaves to generate the laminar and determinate phyllode. From the *Ruscus* phylloclade transcriptome data, we observe that well-studied genes in developmental axes show considerable variation in their presence/absence and paralog copies. As an example, the *AS1* gene of *Arabidopsis thaliana*, also called *RS2* (*Zea mays*) and *PHAN* (*Antirrhinum majus*) [18,19,20], forms a clade with three genes in *Ruscus aculeatus,* three genes in *Sansevieria subspica* (within a closely related genus to *Ruscus* in the tribe Nolinoideae) and one gene in *Oryza sativa* (with a bootstrap support 0.92) (Figure 6). With the high sequence similarity within the clade, all three homologs in *Ruscus aculeatus* were deemed as *AS1-like*.

**Figure 7:**
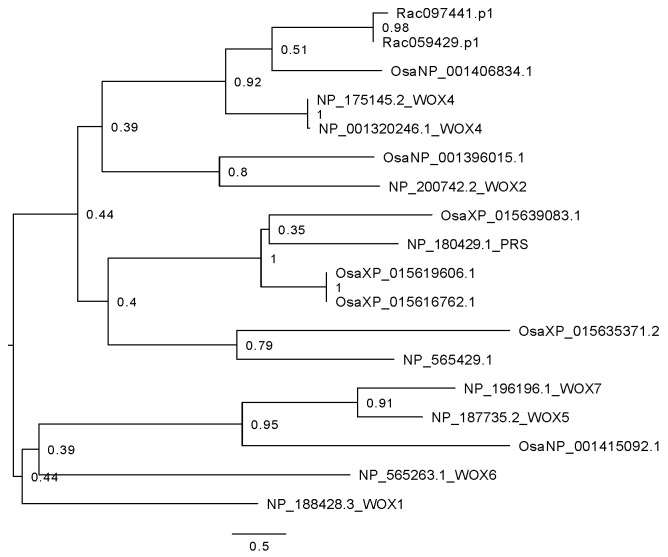
Phylogenetic tree of *WOX* gene families in *Arabidopsis thaliana* (gene name added after the accession number), *Oryza sativa* (Osa), and *R. aculeatus* (Rac). A three-letter species abbreviation is added at the beginning of each gene except for *Arabidopsis thaliana.* Bootstrap values are shown in percentages. No homologs were detected in *Sansevieria subspica* (Ssp).

Some developmental axes genes do not have ortholog families in *R. aculeatus. WOX1*, *WOX2* and *WOX3* (*PRS*) in *Arabidopsis thaliana* are reported to be instrumental in leaf margin development [21]. Their expression is restricted to the interface of adaxial and abaxial domains at the edges of the leaf where they support cell division [16,22,23]. No transcripts were found for *WOX1*, *WOX2*, or *WOX3* in either *R. aculeatus* or *Sansevieria subspica*, whereas a single *WOX3* homolog in *Oryza sativa* is detected. *WOX1* is reported to have been lost in monocots [24], which is consistent with our results concerning this gene. The absence of *WOX 1*, *2*, and *3* is noteworthy, as *WOX1* and *WOX3* specifically have been identified as genes expressed in the leaf margin and involved in margin expansion [16,23,25]. *WOX4* does appear to have two *R. aculeatus* paralogues. Two transcripts in *R. aculeatus* clustered within the *WOX4* clade with a 92% bootstrap support (Figure 7). However, *WOX4* in *Arabidopsis thaliana* cannot compensate for the loss of *WOX1* or *WOX3* [26], and therefore was not included in our subsequent analysis. Because of the nature of our transcriptomic data, we cannot conclude whether the absence of *WOX3* expression in both species (*R. aculeatus* and *S. subspica*) in Nolinoideae is due to gene loss or transcriptional regulation at this time. Other genes without ortholog families include *ARF4*, *CUC*, *MP*, *NGA*, and *TCP*. The results from the remaining targeted genes are summarized in Table 2. The gene trees are given in Supplementary Figures S2–S12.

### 2.6. Generally Low Expression of Developmental Axes Genes in Phylloclade Compared with Stem Tissues

**Figure 8:**
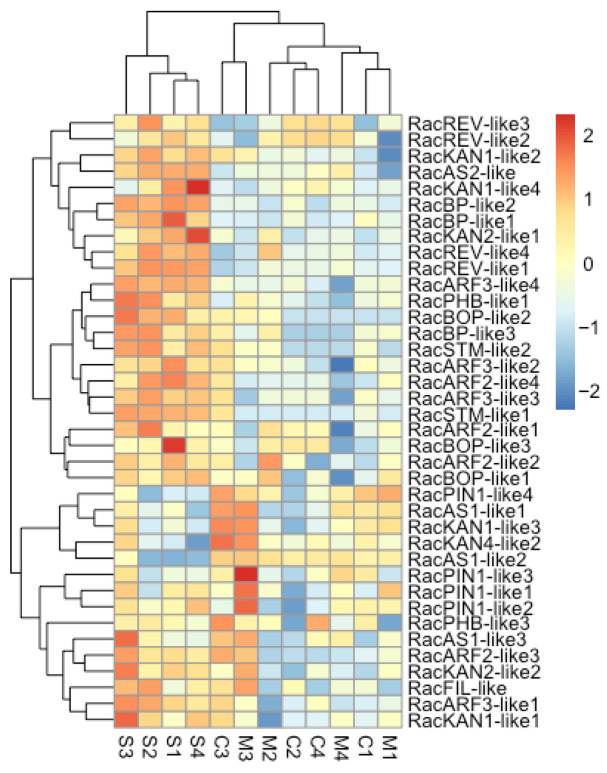
Clustering of gene expression in developmental-axes genes. The developmental-axes genes in *R. aculeatus* were inferred based on their corresponding phylogenetic trees and detailed in Table 2. The rows represent identified gene paralogues in *Ruscus*. The columns represent the individual tissue samples; S1–4 are stem sample replicates, M1–4 are phylloclade margin sample replicates, C1–4 are phylloclade center replicates. Y axis hierarchical groupings reflect correlation of gene expressions across tissues, while X axis hierarchical groupings reflect correlation of sample expression patterns across genes. The continuous color scale in the heatmap represents relative expression values.

We wished to explore the expression patterns of those genes expected to be required for laminar leaf development. To do so, tissue samples and genes were clustered based on correlation of expression patterns of the *Ruscus* contigs listed in Table 2 (Figure 8). The expression analyses of the developmental genes in *R. aculeatus* show a clear clustering of all stem tissue samples into a single group, whereas phylloclade center and margin samples are intermixed. This indicates that phylloclade expression patterns for these genes can be differentiated from those of the stem. However, there is no significant differentiation between center and margin tissue in these canonical leaf gene expressions detected.

Of the putative *Arabidopsis* orthologues represented in Figure 8, all, with the exception of *STM*, were expected to have major roles in the establishment of adaxial/abaxial, proximal/distal, and medial/margin polarities in the leaf and were therefore expected to show increased expression in the phylloclade versus the stem, assuming that the same developmental regulatory modules are utilized to form the phylloclade. The expression pattern of these genes organizes the genes into two major clusters. The first cluster groups four contigs, *RacAS1-like 1*, *RacAS1-like 2*, *RacKAN1-like 3*, and *RacKAN4-like-2*, which show a reduced expression in the stem tissue compared to that in the phylloclade. The second major cluster shows the generally opposite pattern where the genes are of high expression in the stem over in the phylloclade. Based on these results, we see little evidence that canonical leaf adaxial and abaxial modules are activated in the phylloclades that were sampled. One exception may be the recruitment of the *STM/AS1* module to allow initiation of organ differentiation in the phylloclade as a lateral organ. *STM* (*KNOTTED1*) is necessary to maintain indeterminate stem cells in the shoot apical meristem (SAM) and peripheral regions and is repressed by *AS1* (*PHAN*) to allow differentiation in the leaf [27,36]. *RacSTM-like 1* expression is indeed almost absent from the phylloclade but is relatively highly expressed in the stem, while *RacAS1-like 1* and *RacAS1-like 2* display a reciprocal pattern. The increased phylloclade expression of the two KAN paralogues may suggest an abaxial identity, but further *KAN* paralogues of *ARF* contigs expressions are not elevated.

### 2.7. RT-qPCR Comparisons with Transcriptome Data Are Generally Consistent Though Do Demonstrate Biological Sample Variation

**Figure 9:**
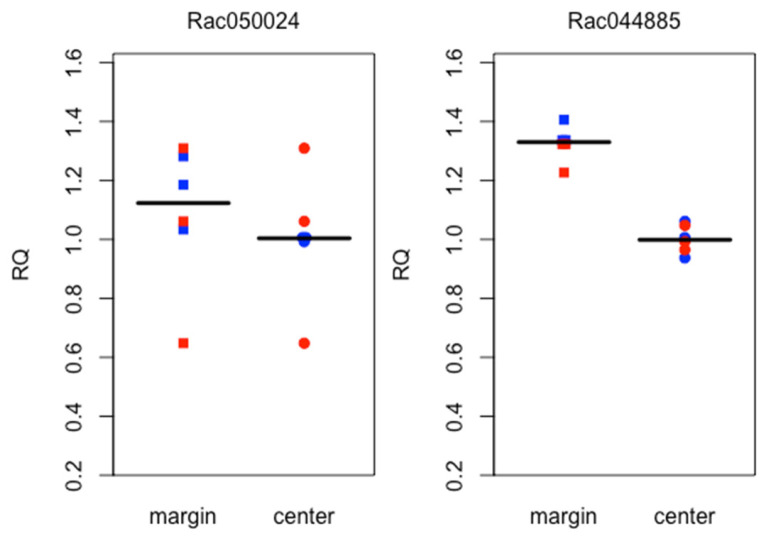
qPCR validation of two selected genes (Rac050024.p1 and Rac044885.p1) exhibiting differential expression between the margin and the center (shown in Figure 3). There are two biological replicates (one shown in blue and one in red) for each gene. Each biological sample includes three technical replicates. The qPCR RQ (relative quantification) values are normalized to the mean of the three technical replicates from the center tissue of the same biological sample. For visualization purposes, the median of all replicates is shown as a horizontal bar.

**Figure 10:**
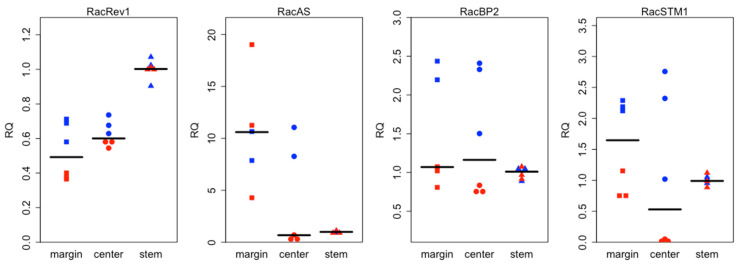
qPCR validation of four selected genes (shown in Figure 8) associated with axial development. As per Figure S1, there are two biological replicates (one shown in blue and one in red) for each gene. Each biological sample includes three technical replicates. The qPCR RQ (relative quantification) values are normalized to the mean of the three technical replicates from the stem tissue of the same biological sample. For visualization purposes, the median of all replicates is shown as a horizontal bar.

Given the expression patterns demonstrated in the transcriptome data and the inherent variation seen among biological samples, we analyzed new biological samples from phylloclade and stem tissue using RT-qPCR against a limited sampling of target genes. To compare between phylloclade margin and center tissue expression patterns, we chose two genes, *Rac050024* and *Rac04885* that had indicated higher expression in the margin tissue compared to that in center tissue in our transcriptome data. To compare between phylloclade and stem expression, we selected four genes that show differential expression between stem and phylloclade tissues in the transcriptome data, *RacREV-like2, RacBP-like2*, *RacAS2-like,* and *RacSTM-like1.* As with the transcriptome data, the RT-qPCR data demonstrated variation among the biological samples of the phylloclade tissue, again perhaps due to differences in development, age, and/or physiological activity at the time of sampling. However, the results were largely consistent with the prior results. Both *Rac04885* and *Rac05024* were consistently overexpressed in the phylloclade margin tissue compared with that of the phylloclade center (Figure 9). *Rac050024* readings varied greatly among samples, though notably, this is consistent with the high standard error recorded for this gene in the transcriptome data. Also, in the stem/phylloclade comparison, *RacREV-like2* showed much higher expression in the stem tissue compared with either phylloclade tissue, and *RacAS2-like* demonstrated higher expression in both phylloclade tissues compared with stem tissue both similar to the transcriptome results. In contrast to the transcriptome results, *RacBP-like2* was highly variable, showing equal or lower expression in the stem compared to phylloclade tissues. *RacSTM-like1* was also highly variable. Consistent with the transcriptome data, it was expressed in very low levels in one central phylloclade tissue, and showed higher expression in the stem tissue (Figure 10). However, contrasting the transcriptome data, its relative expression was higher in the phylloclade margin tissue than in the stem.

## 3. Discussion

Phylloclades in *Ruscus* are functionally analogous to true leaves, with both developing as a determinate planar structure while being derived from axillary primordia and serving as the primary photosynthetic organs. Thus, the study of gene expression, primarily of the growth axis developmental network can identify whether such genes found in leaves are co-opted for phylloclade development. Our results indicate that each tissue tested could be differentiated by the DEGs identified across tissue types (Figure 2 and Figure 5). Notably, the phylloclade center tissue demonstrated enhanced biological functions that would be anticipated with its proposed enhanced photosynthetic activity in comparison with stem and phylloclade margin tissues (Figure 4). Consequently, these comparisons do identify phylloclade as having distinct gene expression patterns (Figure 5). However, when focusing on leaf developmental axes or boundary genes (Table 2), we found that the vast majority are either absent (*ARF4*, *CUC*, *MP*, *NGA*, *TCP*, *WOX 1*, *WOX 2*, and *WOX 3*) or downregulated in comparison with expression found in the cylindrical stem internode (Figure 6, Figure 7 and Figure 8). These results may reflect the lack of strong morphological adaxial/abaxial differentiation in the *Ruscus* phylloclade and are consistent with gene expression studies in other species with cylindrical or unifacial leaves as discussed below.

### 3.1. Ruscus Phylloclade Shows No Consistent Adaxial and Abaxial Differentiation in Morphology and Development

*Ruscus aculeatus*, as well as its congeners *R. hypoglossum* and *R. hypophyllum,* is distinguished by the formation of planar phylloclades or laminar leaf analogs that develop from axillary primordia of leaf scales [4,6,7]. The axillary primordia originally exhibit radial symmetry but then expand medially along the stem, sandwiched between the leaf primordium and the stem, generating a dorso/ventral-like symmetry. The proximal-distal growth of the phylloclade is initiated by apex cells in the center of the primordium and initially grows as a radially symmetric structure similar to the “vorlaüfespitze” or precursor tip found in other monocots such as *Sansevieria* spp. and *Acorus* spp. [37]. The lower part of the phylloclade then grows as a laminar structure from the basal initials that are oriented on the rounded stem. As a result, phylloclades often have a concave shape and may have a ventral side keel opposite the midrib. The phylloclades therefore appear to have a clear top or dorsal side that is adaxial and a distinguishable bottom or ventral (abaxial) side [7,38].

However, other structures and developmental patterns do not indicate clear adaxial/abaxial identities. The epidermal cells on both faces of *R. aculeatus* phylloclades are identical [38]. Cross sections of phylloclades do not show an adaxial/abaxial mesophyll-like structure [4,6,7], and measures of tissue composition, arrangement, and thickness show no significant adaxial/abaxial differences [8]. A subepidermal layer of photosynthetic parenchymal cells surrounds a hydrenchyma, drought tolerant, cortex [8,38]. In all three *Ruscus* species, the primary vasculature develops from the base of the phylloclade as a ring of vessels surrounded by sclerenchyma tissue. The vascular bundles are arranged radially with the xylem facing outward. In *R. aculeatus*, the bundles separate after reaching the bract on fertile phylloclades with parallel secondary bundles on either side of the midvein. Notably, the majority of the bundles are oriented with the xylem facing the axillary leaf or toward the abaxial side as opposed to the common adaxial orientation [7]. This orientation is not fixed as some vessels may be inverted [4,6,7], perhaps the result of flattening the original radial orientation [38]. Similarly, within vascular cylinders in the midvein and general secondary vein, abaxial xylem orientation within individual vascular bundles are found in *R. hypoglossum* and *R. hypophyllum* [7]. In one cross-section of the midvein of *R. hypophyllum*, Arber draws a structure similar to an inverted tri-arc stele that is reminiscent of the stele structure found in the needle-like leaves in *phabulosa* mutants [9].

Placement of reproductive structures also indicates a lack of strict polar identity. In fertile phylloclades in all three *Ruscus* species, bracts and axillary flowers form on the surface of the phylloclades. Generally, the location of the bracts, flowers and subsequent fruit are found on the adaxial or dorsal surface of the phylloclade in *R. aculeatus* and *R. hypoglossum* [4,7]. In contrast, the bracts, flowers, and fruits form primarily on the abaxial side in *R. hypophyllum* [7]. However, in all three species, bract and bud formation may occur on either surface [4,7]. Arber [7] reported two fertile phylloclades from a single node having flower buds on opposite surfaces. Thus, based on epidermal, mesophyll, vascular, and reproductive tissues, *Ruscus* phylloclades do not display a bifacial, adaxial/abaxial differentiation.

### 3.2. Adaxial and Abaxial Gene Expression Signatures Are Consistent with a Unifacial Identity in the Ruscus phylloclade

These observations suggest that the laminar *Ruscus* phylloclades develop as unifacial rather than bifacial structures [37] and so are analogous to the unifacial leaf development found in *Sansevieria*, *Acorus*, and *Juncus* [37,39,40,41,42,43]. If this is true, then we would either expect that the canonical leaf adaxial/abaxial identity genes would not be expressed or would not be functioning as found in model dicot species. Consistent with the former expectation, homologues of the canonical leaf development genes are all significantly downregulated in the phylloclade that we studied relative to their expression in the stem tissue. The presence of their expression in the stem tissue per se is not surprising as the adaxial and abaxial modules composed by *PHB* and *AS1/2* and by *KAN, ARF3/4* and *FIL* respectively are also involved in establishing radial polarity in the stem, root and embryo [44,45,46]. These observations from other species lead us to interpret the higher levels of expression of the canonical adaxial/abaxial genes in the stems of *Ruscus aculeatus* to reflect a reduced expression pattern of these genes in the phylloclade rather than increased expression in the stems. This interpretation needs to be taken with caution as future in situ analyses of gene expression in these tissues at several developmental stages will be necessary to resolve these patterns.

While we could not detect distinctive adaxial/abaxial gene expression patterns in the *Ruscus* phylloclade, there is an indication that the genetic network that suppresses indeterminate and undifferentiated stem cell growth to allow differentiated and determinate lateral organs to form may be active. *AS1* and *AS2* are required in *Arabidopsis* to suppress the expression of *STM* and other *KNOTTED1* family genes [27,36]. *STM* and related genes act to maintain the proliferation of undifferentiated cells in the SAM [27,47]. In *R. aculeatus, ASI/2* orthologues, *RacAS1-like* and *RacAS2-like*, are among the few leaf identity transcripts to be overexpressed in the phylloclades compared to their expression in stems as indicated in both transcriptome and RT-qPCR data sets. In comparison, *RacSTM-like 1* and its paralogue *RacBP* have a reciprocal expression pattern in the transcriptome data with low or no expression in phylloclade tissue, but with strong discernible expression in the stem. Hirayama and colleagues [5] similarly reported low expression of *STM* (*KNOX1*) as well as of *YABBY1* in the phylloclades of *R. aculeatus.* This pattern, however, is less clear in the RT-qPCR data as there is large variation among biological samples of phylloclade tissue. In one sample, *RaSTM-like1* is barely detectable in phylloclade central tissue, but is detected in margin tissues. *RaBP-like2* also is detected in some phylloclade tissues and showed variation among biological samples resulting in similar mean expressions to stem tissue across samples. Secondary expression of *STM* and *BP* in leaves, however, is associated with the formation of lobes in simple leaves and leaflets in compound leaves [48,49]. As such, the variation in expression patterns in phylloclades found in both RT-qPCR and transcriptome analyses may reflect physiological or developmental age variation when the tissues were harvested for study. Again, future in situ hybridization studies over a series of developmental time points may resolve this issue.

## 4. Materials and Methods

### 4.1. Sample Collection and Transcriptome Sequencing

**Figure 11:**
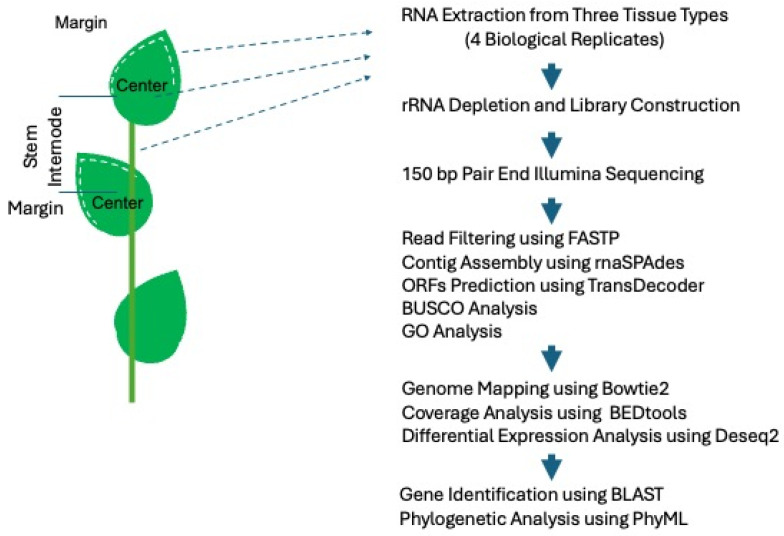
Graphic flowchart of experimental methods. Tissues from the phylloclade margins and center and from the stem internode were dissected. Total RNA was extracted from the tissue from which a tRNA selected library was prepared for RNAseq. The raw sequence data was then trimmed and filtered for high-quality reads. The reads were then assembled into contigs and ORFs were predicted. These were then submitted for BUSCO analysis to determine the coverage quality and GO analyses were executed to identify enhanced functional categories for each tissue. DEGs were identified and gene identities were predicted through BLAST 2.13.0 searches and gene phylogenetic analyses.

Individual plants of *Ruscus aculeatus* “Elizabeth Lawrence” were purchased from Plant Delights Nursery, Raleigh, NC, USA. Total RNAs were extracted from tissue 1–2 mm from the margins and from center tissue of expanding young, pre-sclerified but formed, phylloclades, at or near the top of young secondary branches (Figure 11), and from adjacent internode using the Zymo Quick RNA Plant kit following the manufacturer’s protocol. Four samples were collected for each tissue type (i.e., C1, C2, C3, C4 for center tissue, M1, M2, M3, M4 for margin tissue, S1, S2, S3, S4 for stem tissue). Sequencing libraries were constructed from rRNA-depleted RNA pools and sequenced by AZENTA (South Plainfield, NJ, USA) using standard RNA-seq protocol producing paired 150 bp reads (details in Table 1).

### 4.2. Sequence Analyses

Genome assembly was constructed following our previous study [39]. Raw RNA-seq reads (paired-end 150 bp) were processed using the FASTP program [50] to filter low-quality data. Filtered sequencing reads were assembled into contigs using rnaSPAdes [51]. Samples of the center and marginal phylloclade tissue and internode tissue from the same plant were merged to build reference contigs to include any tissue-specific transcripts. Open reading frames (ORFs) were predicted using the TransDecoder program in Trinity [52]. Raw sequencing reads were mapped to the corresponding assembled contigs using Bowtie2 [53]. The completeness of the transcriptome assembly was evaluated using BUSCO v6.0.0 [54] using the *embryophyta_odb12* lineage dataset (1 July 2025). Read coverage for each annotated ORF was generated using BEDtools [55]. Differential gene expression between center and margin tissues was determined using DEseq2 [56]. Normalized gene expression was plotted into heatmaps using the R package Pheatmap (DOI:10.32614/CRAN.package.pheatmap).

### 4.3. Analysis of Genes in Developmental Axes

We analyzed gene expression in *Ruscus aculeatus* of 18 developmental axes genes, all of which have been identified to play significant roles in leaf development [13,14,15]. To identify *R. aculeatus* homologs of these genes, amino acid sequences of each target protein were selected from *Arabidopsis* (GenBank accession GCA_000001735.2) and used in BLAST searches against *Oriyza sativa* (GenBank accession GCA_034140825.1). These sequences were then used in BLAST searches against the *R. aculeatus* transcriptome contigs generated in this study and against the transcriptome of *Sansevieria subspica* generated in a previous study [39]. *S. subspica* is within a closely related genus to *Ruscus*, both within the same subfamily Nolinoideae. Putative homologous protein sequences were aligned using MUSCLE [57]. Phylogenetic trees were constructed based on their amino acid sequences using PhyML [58] under a JTT + Γ substitution model. Based on their proposed phylogenetic relationship to the *Arabidopsis* gene, *R. aculeatus* contigs were then back-BLASTed to the *Arabidopsis* genome to confirm that the contig represents a likely homologue. When no *R. aculeatus* contig could be identified through gene tree or BLAST analyses, we did not include such genes in further analyses.

### 4.4. RT-qPCR Analysis of Selected Genes

We selected six genes from those identified as DEGs in the transcriptome analysis, either demonstrating differentiation between phylloclade margin and center or between the phylloclade and stem, *Rac05024, Rac04885*, *RacREV-like2, RacBP-like2*, *RacAS2-like,* and *RacSTM-like1*. Additionally, the sequence in our transcriptome assembly demonstrating the highest similarity to the Ubiquitin-specific protease (*UBP)* in a BLAST search was used as an expression standardization gene. Primers were designed using Primer3 software to target 100 to 250 bp fragments from these genes, based on sequences from our transcriptome assembly. A list of the genes and corresponding primer sequences are given in Supplemental Table S2. Verification of the primers to amplify single gene products was accomplished by executing standard PCR reactions using *R. aculeatus* genomic DNA as a template and Promega GoTaq Master Mix. Genomic DNA was isolated from fresh tissue using the Zymo Plant/Seed Mini Kit following manufacturer protocols. The reaction conditions were 96 °C for 2 min followed by 30 cycles of 96 °C for 20 s, 57 °C for 20 s and 72 °C for 30 s. A 10 µL aliquot from each reaction was then electrophoresed on a standard 1.5% TBE Synergel/Agarose gel and stained with ethidium bromide to assess the presence and size of the reaction products. Apparent single gene products were later verified by the presence of single peaks on melting point curves following qPCR reactions on the Applied Biosystems QuantStudio3.

All qPCR reactions were assembled using NEB Luna Universal qPCR Master Mix following the manufacture protocol. Standard curves and amplification efficiencies for each primer pair were assessed using a 1 to 4 serial dilution of *R. aculeatus* genomic DNA with a starting concentration of approximately 60 ng/µL. Batch reactions were prepared for each primer pair, then aliquoted into four sub-batches, each of which contains a different dilution of template DNA. These were then divided into three 10 µL technical replicates. Reactions were run on an Applied Biosystems QuantStuio3 with settings of 94 °C for 10 min followed by 40 cycles of 95 °C for 15 s, 57 °C for 30 s, and 60 °C for 30 s. Amplification efficiencies were estimated using the QuantStudio3 DA3 software.

Total RNA was extracted from phylloclade and stem internode tissue using the Zymo Plant/Seed Mini RNA kit as described earlier. Each sample was treated with DNaseI for 15 min at room temperature and then cleaned and concentrated using NEB Monarch RNA Cleanup columns following the manufacturer’s protocol. RNA was quantified using a Nanodrop spectrophotometer. cDNA was generated using the NEB LunaScript RT SuperMix following the manufacturer protocol.

As described above, all RT-qPCR reactions were assembled using NEB Luna Universal qPCR Master Mix following the manufacture protocol. To ensure equal loading of tissue-specific cDNA across gene-specific reactions, batch reactions with cDNA from each tissue were mixed without primers, then aliquoted to sub-batches into which specific gene primers were added. Specifically, for each cDNA template, 122.5 µL Luna qPCR Master Mix, 73.5 µL H_2_O, and 24.5 µL cDNA template were mixed and aliquoted into seven tubes with 31.5 µL each. 3.5 µL of a primer solution containing 10 µM of both forward and reverse primers was added for each specific gene target. After mixing by pipetting, each reaction was further aliquoted into three 10 µL technical replicates per cDNA/gene reaction in a 96-well plate. The reaction conditions were set as above. All data analyses were done using the Applied Biosystems QunatStudio3 DA3 software.

## 5. Conclusions

The formation of laminar phylloclades in the apparent absence of transcriptionally defined adaxial and abaxial tissue suggests that alternative developmental pathways may result in planar structures. The loss of adaxial and abaxial juxtaposition in margins could be used to describe the development of ensiform or cylindrical leaf formation as derived characters from ancestral planar leaves such as seen in the monocots *Acorus*, *Sansevieria*, or *Juncus*. However, in the case of phylloclades in *Ruscus*, the planar and determinate structures have been interpreted most parsimoniously as being derived from ancestral cylindrical, indeterminate branches. In this case the laminar structures appear to develop without strong adaxial and abaxial identities. Whether this pattern of unifacial (single polarity) development reflects an ancestral condition in monocots or basal angiosperms or a derived loss of a developmental network is unclear. Further transcriptional studies in unifacial monocot and basal angiosperm leaves will be necessary to determine whether the loss of adaxial and abaxial gene activities in leaves is lineage specific or whether the gain of activity of such genes in unrelated eudicot and monocot species is an example of convergent evolution. As discussed above, many of these genes are also expressed within the vascular bundles in stem and root tissues and appear to be involved in radial patterning. A co-opted, derived utilization of these genes in establishing leaf polarity is therefore plausible.

The vast variation in leaf morphology in angiosperms and the extensive homology of upstream, developmental regulators provide an expansive opportunity to explore the evolutionary trajectories of complex organs [59]. Monocots, in general, tend to be isobilateral in their structure and form. While exceptions do occur, there tends to be little to no dorsal/ventral identity differentiation in monocot leaves in comparison with eudicot leaves. Leaf epidermis tends to be either identical or similar with stomata commonly found on both surfaces. Similarly, mesophyll tissue in monocots tends not to differentiate in an adaxial/abaxial polarity as it generally does in eudicots. These generalizations suggest that the networks that establish adaxial and abaxial identities may not be active or dominant during monocot leaf development. It must be recognized that this study is based on one or several sequentially close time points. Investigation utilizing multiple developmental stages as well as more spatially defined in situ hybridization studies will likely be more informative. Additionally, expanding and testing these developmental models across the morphological diversity of non-model plant species will further elucidate the complexity and plasticity of these regulatory networks.

## Figures and Tables

**Table 1:** Basic information of the transcriptomic data.

Sample	Tissue Type	Raw Reads	Reads After Filtering	Mapped Reads (%)
Phylloclade Tissue				
C1	Center	22,172,852	21,657,091	95.59
C2	Center	22,531,329	21,987,593	96.93
C3	Center	22,377,207	22,843,938	95.65
C4	Center	18,319,144	17,843,690	95.80
M1	Margin	17,656,322	17,262,433	94.40
M2	Margin	18,347,774	17,936,262	96.50
M3	Margin	20,192,908	19,640,406	95.57
M4	Margin	22,499,088	21,870,150	95.67
Stem Tissue				
S1	Stem	21,098,577	20,560,206	95.59
S2	Stem	19,295,352	18,852,449	96.53
S3	Stem	21,332,822	20,828,960	95.31
S4	Stem	19,593,931	19,055,362	95.41

**Table 2:** Developmental axes genes in *R. aculeatus*. Genes are grouped roughly by adaxial/abaxial identity and by meristemic regulation, either positive or negative. Some genes may potentially be placed in alternative groupings as their expression patterns may overlap in function and position identity.

Gene	No. of Orthologs in *Ruscus*	Gene Name	Assigned Name
ADAXIAL			
*AS1* [27]	3	Rac011161.p1 Rac058882.p1 Rac033888.p1	*RacAS1-like1* *RacAS1-like2* *RacAS1-like3*
*AS2* [27]	1	Rac040075.p1	*RacAS2-like*
*PHB/PHV* [10]	4	Rac008059.p1 Rac006965.p1 Rac003767.p1	*RacPHB-like-1* *RacPHB-like-2* *RacPHB-like-3*
*REV* [10]	4	Rac003284.p1 Rac005721.p1 Rac005670.p1 Rac002707.p1	*RacREV-like1* *RacREV-like2* *RacREV-like3* *RacREV-like4*
ABAXIAL			
*ARF4* [21]	0		
			
			
*KAN1* [28]	4	Rac027202.p1 Rac031012.p1 Rac029139.p1 Rac030168.p1	*RacKAN1-like1* *RacKAN1-like2* *RacKAN1-like3* *RacKAN1-like4*
*KAN2/3* [28]	2	Rac031620.p1 Rac044466.p1	*RacKAN2-like1* *RacKAN2-like2*
*KAN4* [28]	2	Rac045480.p1 Rac034985.p1	*RacKAN4-like1* *RacKAN4-like2*
MERISTEMIC REGULATION (Positive or Negative/Boundary)			
*BOP1* [29] *BOP2* [29]	3	Rac044824.p1 Rac073619.p1 Rac067630.p1	*RacBOP-like1* *RacBOP-like2* *RacBOP-like3*
*BP* [30]	3	Rac031580.p1 Rac029107.p1 Rac041665.p1	*RacBP-like1* *RacBP-like2* *RacBP-like3*
*CUC* [31]	0		
*FIL* [32]	1	Rac078913.p1	*RacFIL-like*
*MP* [21]	0		
*NGA* [33]	0		
*PIN1* [34]	4	Rac055077.p1 Rac060767.p1 Rac058126.p1 Rac054963.p1	*RacPIN1-like1* *RacPIN1-like2* *RacPIN1-like3* *RacPIN1-like4*
*STM* [10]	2	Rac036602.p1 Rac086951.p1	*RacSTM-like1* *RacSTM-like2*
*TCP* [33]	0		
*WOX1* [35]	0		
*WOX2* [35]	0		
*WOX3* [35]	0		

## Data Availability

The datasets generated and/or analyzed during the current study are available in the Sequence Read Archive (SRA repository), under the BioProject accession number PRJNA1164170.

## Supplementary Materials

The following supporting information can be downloaded at https://www.mdpi.com/article/10.3390/plants15081168/s1, Supplemental Figure S1. Volcano plot showing gene expression changes (in log2FoldChange) in the comparison between phylloclade margin to phylloclade center (A) and in the comparison between stem and phylloclade (B). Supplemental Figure S2. Phylogenetic tree of *ARF* gene families in *Arabidopsis thaliana* (gene name added after the accession number)*, Oryza sativa* (Osa), and *R. aculeatus* (Rac). A three- letter species abbreviation is added at the beginning of each gene except for *Arabidopsis thaliana.* Bootstrap values are shown in percentages. Supplemental Figure S3. Phylogenetic tree of *BOP* gene families in *Arabidopsis thaliana* (gene name added after the accession number)*, Oryza sativa* (Osa), and *R. aculeatus* (Rac). A three- letter species abbreviation is added at the beginning of each gene except for *Arabidopsis thaliana.* Bootstrap values are shown in percentages. Supplemental Figure S4. Phylogenetic tree of *CUC* gene families in *Arabidopsis thaliana* (gene name added after the accession number)*, Oryza sativa* (Osa), and *R. aculeatus* (Rac). A three- letter species abbreviation is added at the beginning of each gene except for *Arabidopsis thaliana.* Bootstrap values are shown in percentages. Supplemental Figure S5. Phylogenetic tree of *FIL* gene families in *Arabidopsis thaliana* (gene name added after the accession number)*, Oryza sativa* (Osa), and *R. aculeatus* (Rac). A three- letter species abbreviation is added at the beginning of each gene except for *Arabidopsis thaliana.* Bootstrap values are shown in percentages. Supplemental Figure S6. Phylogenetic tree of *KAN* gene families in *Arabidopsis thaliana* (gene name added after the accession number)*, Oryza sativa* (Osa), and *R. aculeatus* (Rac). A three- letter species abbreviation is added at the beginning of each gene except for *Arabidopsis thaliana.* Bootstrap values are shown in percentages. Supplemental Figure S7. Phylogenetic tree of *MP* gene families in *Arabidopsis thaliana* (gene name added after the accession number)*, Oryza sativa* (Osa), and *R. aculeatus* (Rac). A three- letter species abbreviation is added at the beginning of each gene except for *Arabidopsis thaliana.* Bootstrap values are shown in percentages. Supplemental Figure S8. Phylogenetic tree of *PIN1* gene families in *Arabidopsis thaliana* (gene name added after the accession number)*, Oryza sativa* (Osa), and *R. aculeatus* (Rac). A three- letter species abbreviation is added at the beginning of each gene except for *Arabidopsis thaliana.* Bootstrap values are shown in percentages. Supplemental Figure S9. Phylogenetic tree of *PHB/REV* gene families in *Arabidopsis thaliana* (gene name added after the accession number)*, Oryza sativa* (Osa), and *R. aculeatus* (Rac). A three-letter species abbreviation is added at the beginning of each gene except for *Arabidopsis thaliana.* Bootstrap values are shown in percentages. Supplemental Figure S10. Phylogenetic tree of *NGA* gene families in *Arabidopsis thaliana* (gene name added after the accession number)*, Oryza sativa* (Osa), and *R. aculeatus* (Rac). A three- letter species abbreviation is added at the beginning of each gene except for *Arabidopsis thaliana.* Bootstrap values are shown in percentages. Supplemental Figure S11. Phylogenetic tree of *STM/BP* gene families in *Arabidopsis thaliana* (gene name added after the accession number)*, Oryza sativa* (Osa), and *R. aculeatus* (Rac). A three-letter species abbreviation is added at the beginning of each gene except for *Arabidopsis thaliana.* Bootstrap values are shown in percentages. Supplemental Figure S12. Phylogenetic tree of *TCP* gene families in *Arabidopsis thaliana* (gene name added after the accession number)*, Oryza sativa* (Osa), and *R. aculeatus* (Rac). A three-letter species abbreviation is added at the beginning of each gene except for *Arabidopsis thaliana.* Bootstrap values are shown in percentages. Table S1. Information of 60 DEGs with baseMean > 50 and |log2FoldChange| > 1. (Table S1 is provided as an excel file as Table S1. Information of 60DEGs.xlsx). Table S2. Primer sequences for RT-qPCR analyses.
