# Supplementary material for: Transcriptomic Analysis of Phylloclade in Ruscus aculeatus Is Consistent with Unifacial Morphology
Source: Plants (Basel). 2026 Apr 10;15(8):1168. doi: 10.3390/plants15081168 (PMC13120324; doi:10.3390/plants15081168)
Supplement: Supplementary file 1 [file plants-15-01168-s001.zip › Table S2.pdf]

Table S2. Primer sequences for RT-qPCR analyses.

Rac004919.p1_UBP9

Forward Primer	5′	GTTTAGGGGTTGGGAAGAAT 3′
Reverse Primer	5′	TTCTGCGGAGACTCATTACA 3′

Rac005721.p1_RacREV-like2

Forward Primer	5′	TTGGACTTAGTTCGGGTGAT 3′
Reverse Primer	5′	CACACCATTTTCAACAAGGA 3′

Rac036602.p1_RacSTM-like1

Forward Primer	5′	GAACTACGTTGATCCCCAAG
Reverse Primer	5′	GGGTAAGGCCATTTGTAGTG

Rac040075.p1_RacAS2-like

Forward Primer	5′	AGATCCAGCACGATCTCTTC
Reverse Primer	5′	CTGCTGCTCTCTAAACGACA

Rac029107.p1_RacBP-like2

Forward Primer	5′	TTCCTCCGAAGAAGATCAAG
Reverse Primer	5′	AGCTCCCACCAGTTGAGTAG

Rac044885.p1

Forward Primer	5′	TCAAGGAGAAGGTGATGGAT
Reverse Primer	5′	GCTCCCATCTCACCTGTAAC

Rac050024.p1

Forward Primer	5′	TCTCAAGTGTGGTCCAGATG
Reverse Primer	5′	GCCGGTGTAGAAGGAGTAGA
