# Supplementary figures and images for: Transcriptomic Analysis of Phylloclade in Ruscus aculeatus Is Consistent with Unifacial Morphology
Source: Plants (Basel). 2026 Apr 10;15(8):1168. doi: 10.3390/plants15081168 (PMC13120324; doi:10.3390/plants15081168)

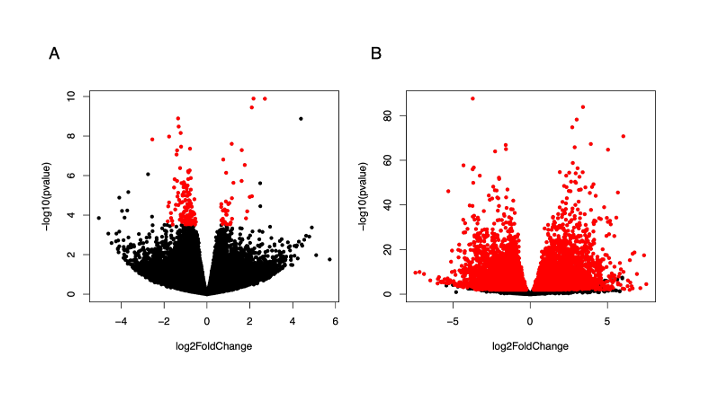

Supplement: Supplementary file 1 [file plants-15-01168-s001.zip › FigS1.tiff]

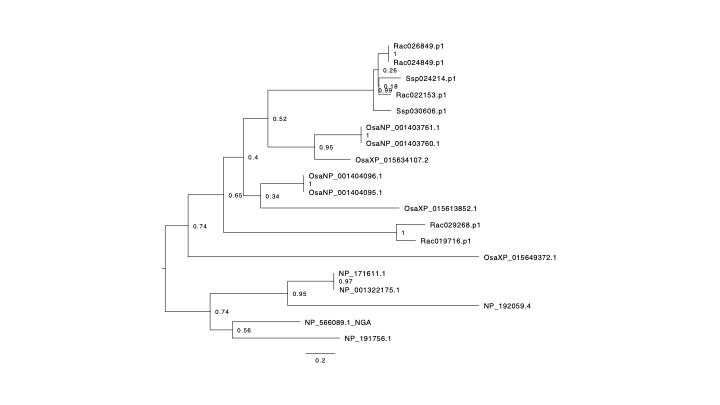

Supplement: Supplementary file 1 [file plants-15-01168-s001.zip › FigS10.tiff]

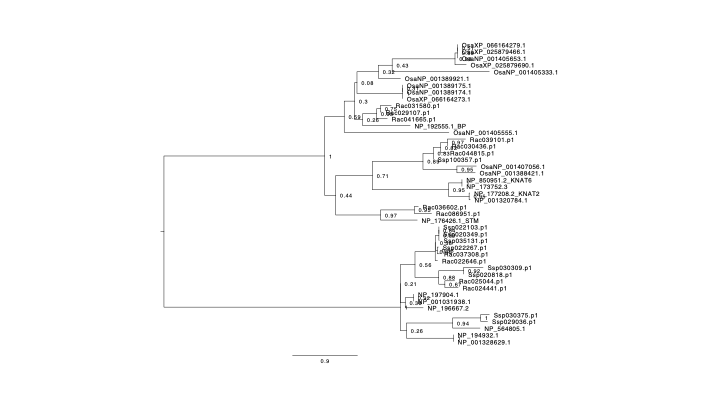

Supplement: Supplementary file 1 [file plants-15-01168-s001.zip › FigS11.tiff]

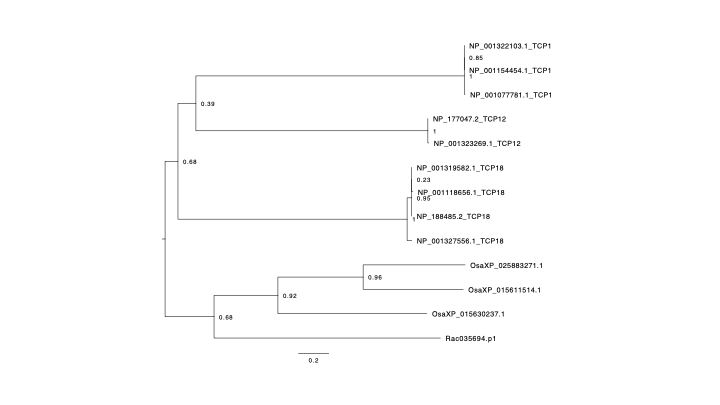

Supplement: Supplementary file 1 [file plants-15-01168-s001.zip › FigS12.tiff]

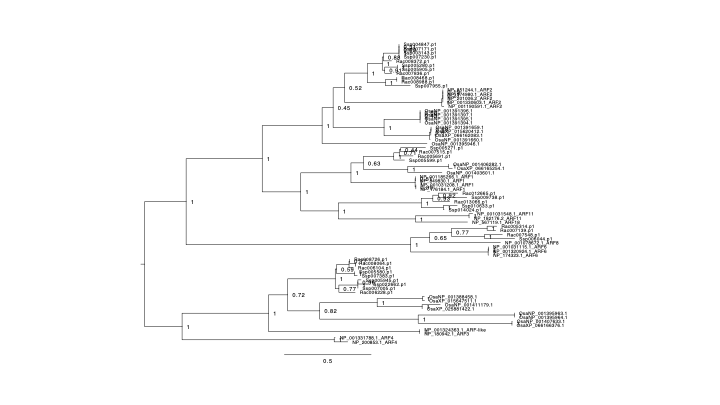

Supplement: Supplementary file 1 [file plants-15-01168-s001.zip › FigS2.tiff]

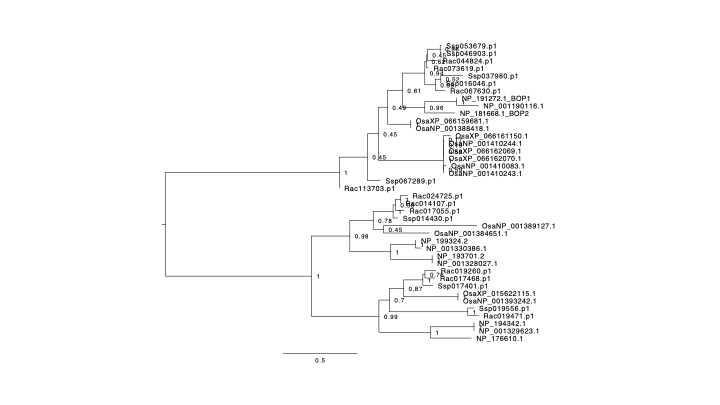

Supplement: Supplementary file 1 [file plants-15-01168-s001.zip › FigS3.tiff]

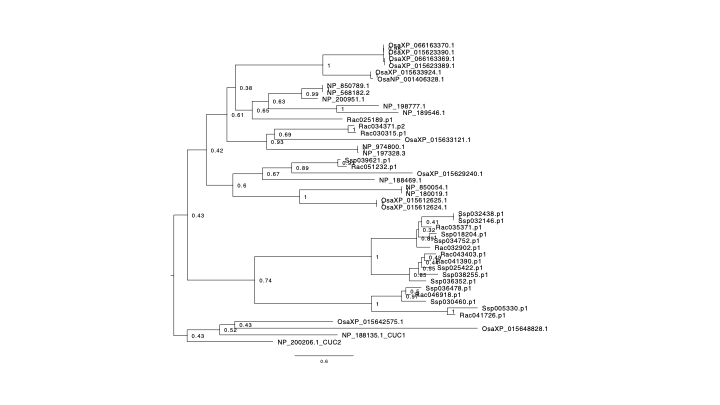

Supplement: Supplementary file 1 [file plants-15-01168-s001.zip › FigS4.tiff]

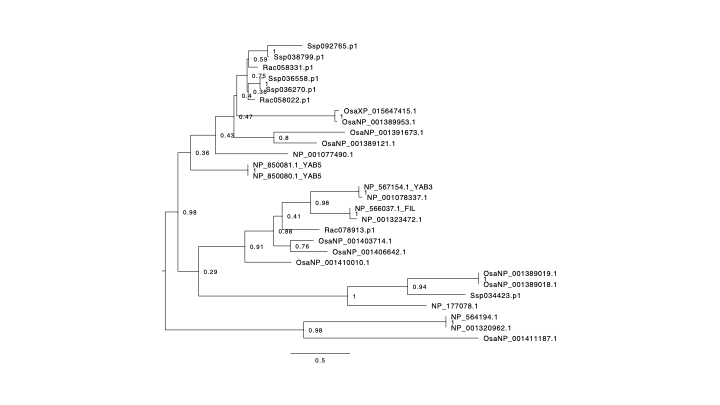

Supplement: Supplementary file 1 [file plants-15-01168-s001.zip › FigS5.tiff]

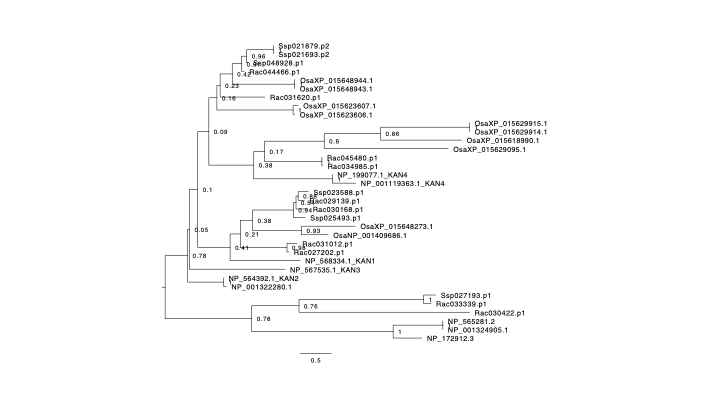

Supplement: Supplementary file 1 [file plants-15-01168-s001.zip › FigS6.tiff]

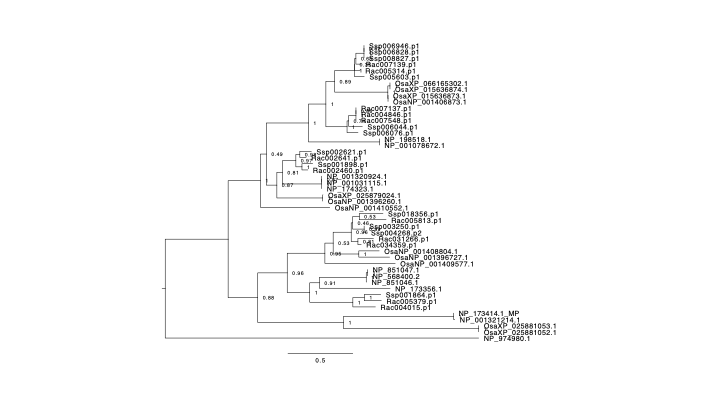

Supplement: Supplementary file 1 [file plants-15-01168-s001.zip › FigS7.tiff]

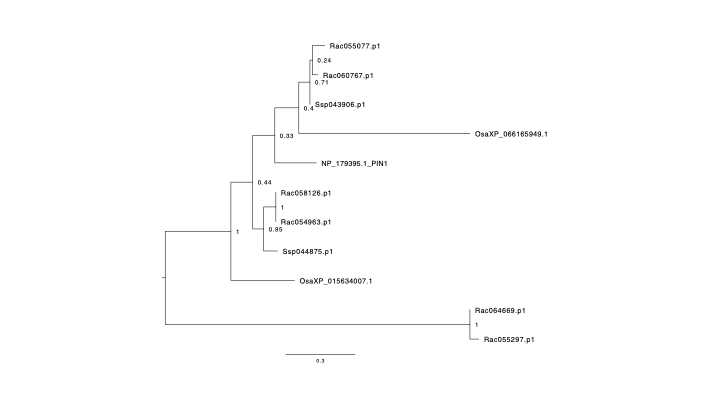

Supplement: Supplementary file 1 [file plants-15-01168-s001.zip › FigS8.tiff]

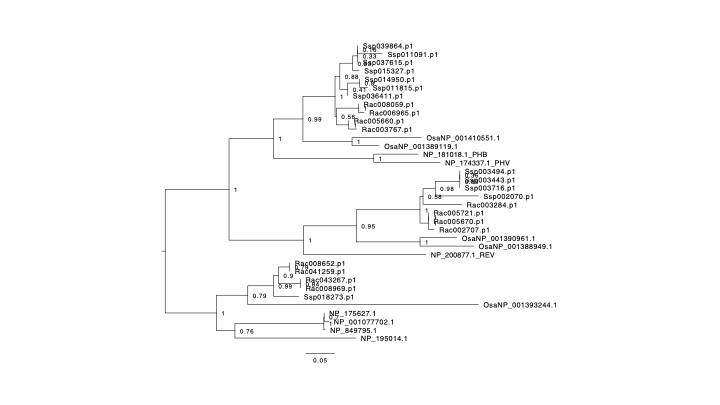

Supplement: Supplementary file 1 [file plants-15-01168-s001.zip › FigS9.tiff]
